# Clinical characteristics and outcome of pediatric patients diagnosed with Langerhans cell histiocytosis in pediatric hematology and oncology centers in Poland

**DOI:** 10.1186/s12885-020-07366-3

**Published:** 2020-09-11

**Authors:** Anna Raciborska, Katarzyna Bilska, Jadwiga Węcławek-Tompol, Olga Gryniewicz-Kwiatkowska, Małgorzata Hnatko-Kołacz, Joanna Stefanowicz, Anna Pieczonka, Katarzyna Jankowska, Filip Pierelejewski, Tomasz Ociepa, Grażyna Sobol-Milejska, Katarzyna Muszyńska-Rosłan, Olga Michoń, Wanda Badowska, Monika Radwańska, Katarzyna Drabko

**Affiliations:** 1 Department of Oncology and Surgical Oncology for Children and Youth, Institute of Mother and Child, Kasprzaka 17a, 01-211 Warszawa, Poland; 2 Department and Clinic of Pediatric Oncology, Hematology and Bone Marrow Transplantation, Wroclaw Medical University, Wrocław, Poland; 3 Department of Oncology, Children’s Memorial Health Institute, Warszawa, Poland; 4 Department of Oncology and Hematology, University Children’s Hospital of Cracow, Kraków, Poland; 5 Department of Pediatric Hematology and Oncology, Medical University of Gdansk, Gdańsk, Poland; 6 Department of Pediatric Oncology, Hematology and Transplantology, Medical University of Poznan, Poznań, Poland; 7 Department of Pediatric Hematology and Oncology Collegium Medicum, Nicolaus Copernicus University, Bydgoszcz, Poland; 8 Department of Pediatrics, Oncology and Hematology, Medical University of Lodz, Łódź, Poland; 9 Department of Pediatrics, Hematology and Oncology, Medical University of Szczecin, Szczecin, Poland; 10 Unit of Pediatric Oncology, Hematology and Chemotherapy, Medical University of Silesia, Katowice, Poland; 11 Department of Pediatric Oncology and Hematology, Medical University of Bialystok, Children’s Clinical Hospital of L. Zamenhof, Białystok, Poland; 12 Department of Pediatrics, Hematology and Oncology, Medical University of Zabrze, Zabrze, Poland; 13 Department of Pediatric Oncology, Hematology, Medical University of Olsztyn, Olsztyn, Poland; 14 Department of Pediatric Oncology, Hematology, Medical University of Rzeszow, Rzeszów, Poland; 15 Department of Pediatric Hematology, Oncology and Transplantology, Medical University of Lublin, Lublin, Poland

**Keywords:** Histiocytosis, Treatment, Survival, Children

## Abstract

**Background:**

Langerhans cell histiocytosis (LCH) affects 1–2 in 1,000,000 people. The disease is not associated with increased risk of treatment failure (especially among older children), but appropriate procedures implemented in advance can eliminate complications which might appear and significantly worsen the patients’ quality of life. Thus, we sought to evaluate the clinical features, management, and outcome of children with LCH treated in Polish pediatric hematology-oncology centers.

**Materials and methods:**

One hundred eighty two patients with LCH were treated according to the Histiocytic Society Guidelines between 2010 and 2017. The participating centers were requested to provide the following data: demographic, clinical, as well as local or systemic treatment data and patients’ outcome. Overall survival (OS) and event free survival (EFS) were estimated by Kaplan-Meier methods and compared using the log-rank test.

**Results:**

Sixty nine percent of children were classified as single system (SS). The patients with SS disease were significantly older as compared to the children with multisystem disease (MS), 6 vs. 2.3 years respectively (p 0.003). Bones were involved in 76% of patients. Systemic treatment was applied to 47% of children with SS disease and 98% with MS disease. Fourteen patients relapsed while two children died. OS and EFS in entire group were 0.99 and 0.91 respectively (with median follow-up 4.3 years).

**Conclusion:**

The treatment of LCH in Polish centers was effective, however, new approaches, including mutation analyses and good inter-center cooperation, are needed to identify patients who might require modification or intensification of treatment.

## Background

Langerhans cell histiocytosis (LCH) affects 5–9 in 10^6^ children younger than 15 years and 1 in 10^6^ older patients. At the origin of the disease lies the clonal proliferation of histiocytes called Langerhans cells. Its symptoms are the result of accumulation of the abnormal cells in tissues and organs. The disease is not associated with increased risk of treatment failure (especially among older children) However, appropriate procedures implemented preemptively can eliminate possible complications and so significantly reduce the risk of deterioration of patients’ quality of life [1–6].

Currently, new approaches and procedures allow to identify the patients requiring modification or intensification of treatment. As part of routine diagnostics, the gene profile and targeted therapies are increasingly used in treatment regimens [7–13].

Unfortunately, the treatment of children with LCH in Poland continues to be limited by financial resources, legal restrictions to introducing new treatment protocols and clinical studies for children, as well as by the lack of national cooperative clinical trials. Owing to the aforementioned reasons, the Polish Children Oncology Group adopted the Histiocyte Society Guidelines for LCH III in 2010. This was the first attempt to unify the management of children with LCH in Poland. In spite of this early effort, none of the Polish oncological centers used randomization at the time. Instead, they determined the treatment arm independently. To our knowledge, there has been no prior clinical assessment of their decision.

Thus, in the present study, we sought to: 1) evaluate the efficacy of the managements adopted by each oncological center on their own, with no inter-center cooperation; 2) assess the outcome of children with LCH treated in Polish pediatric hematology-oncology centers. We believe that the ability to implement complex studies by centers and countries traditionally excluded from large cooperative groups is key to generalize the results of treatment to children with LCH globally.

## Methods

### Patients

Since 1962, by the decision of the Polish Minister of Health, the care for children with cancer is provided separately from adult oncology. Currently, there is a very well-functioning oncology care system for children and adolescents with 18 centers located in major cities throughout the country.

For the purpose of this study, all Polish child oncology centers were requested, based on national regulations, to provide the following data: demographic data, clinical data, local or systemic treatment data and outcome of the patients treated due to LCH between January 2010 and December 2017. As a result, we were able to retrospectively collect data from 14 out of 18 pediatric oncology centers in Poland. All patients had a histological confirmation of LCH at the time of diagnosis using immunohistochemical method without central verification, which was not implemented until 2018. Patients were assessed as single system disease (SS: defined as one organ or system involved) or as multi system disease (MS: defined as two or more organs or systems involved) [6]. The approval for this retrospective study was obtained from all relevant institutions in compliance with national law and international regulations for protection of Human research subjects.

### Statistical methods

Overall Survival (OS) was defined as the time interval from the date of diagnosis to the date of death or to the date of last follow-up. Event-free survival (EFS) was defined as any of the following: the time interval from the date of diagnosis to the date of disease progression, recurrence, second malignancy, death of any reason or to the date of last follow-up for patients without above events. Results distributions were estimated using the method of Kaplan-Meier. Survival curves in groups were compared using log-rank test and *p* ≤ 0.05 was regarded as significant. Statistical analysis was performed using Statistica 13.3 for Windows.

## Results

### Patients characteristic and treatment

One hundred and eighty-two patients with LCH were treated using Histiocyte Society Guidelines [14] during the period between 2010 and 2017. There were 71 (39%) boys and 111 (61%) girls. The majority of children (69%) were classified as single system. Median age at diagnosis was 4.2 years, however, the patients with single system disease (SS) were significantly older as compared to the children with multisystem disease (MS), that is 6 years vs. 2.3 years respectively (p 0.003). The most common site was bones (76% patients with SS). The next most common locations were skin (16%), followed by lymph nodes (3%). Only one patient had isolated CNS involvement. MS disease presented with more than two organs involvement was found in 33 (59%) out of 56 cases [15]. Molecular tests were performed in 26 (14%) of patients. BRAF mutation was found in 11 participants (42%).

Systemic treatment was applied to 112 patients: 47% children with SS disease and 98% with MS disease. One hundred nine children received systemic chemotherapy according to Histiocyte Society Treatment Guidelines adopted by Polish Children Oncology Group [14]. As local therapy, radiotherapy was applied in two cases, and a surgery was performed with or without local steroids in seven patients. The patient clinical and treatment characteristics are shown in Table 1.

**Table 1:** Patient Characteristics

	SS	MS	p
Number of patients	126 (69%)	56 (31%)	
Age			
years: median (range)	6 (0.1–18.1)	2.2 (0.2–16.5)	0.001
Sex			
Male	49 (39%)	22 (39%)	
Female	77 (61%)	34 (61%)	0.89
Chemotherapy			
Yes	59 (47%)	55 (98%)	
No	67 (53%)	1 (2%)	

*SS* Single system, *MS* Multisystem;

### Follow-up and outcome

**Fig. 1:**
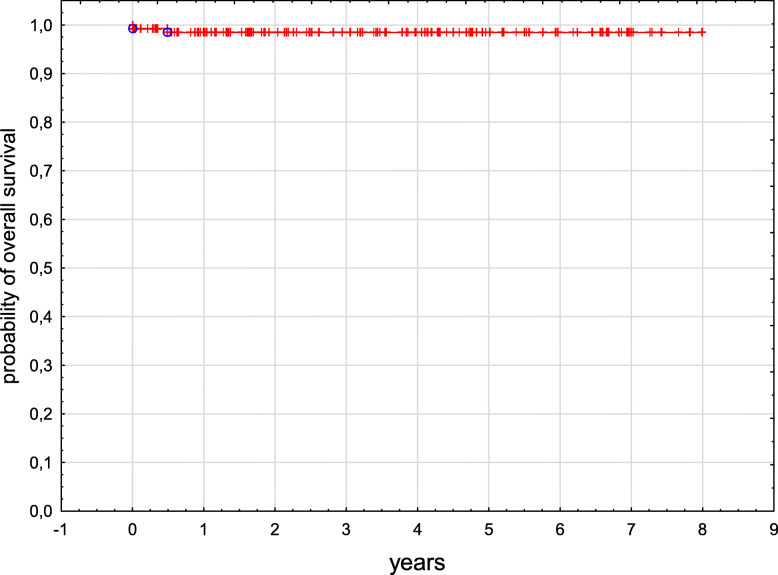
Kaplan-Meier curve of OS for the entire study group

**Fig. 2:**
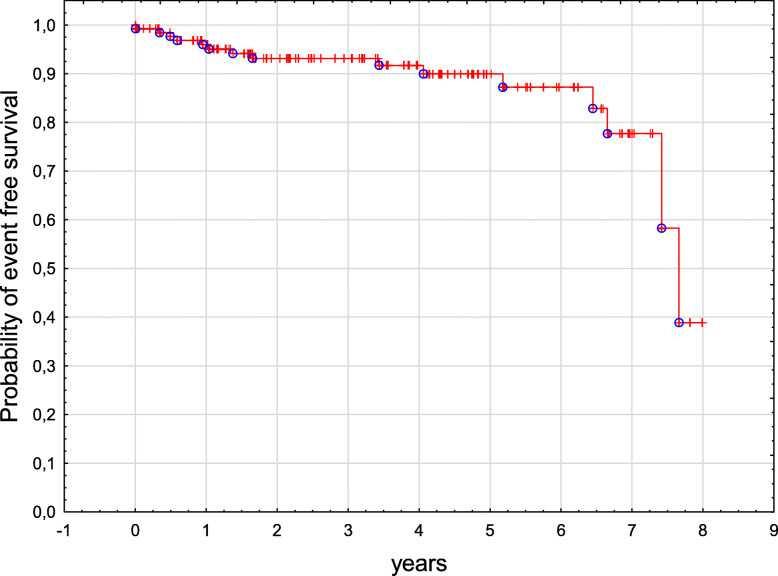
Kaplan-Meier curve of EFS for the entire study group

**Fig. 3:**
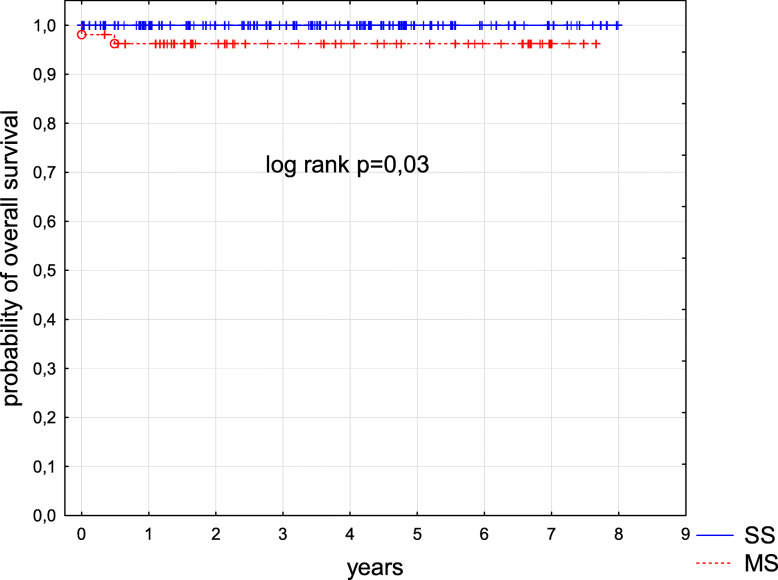
Kaplan-Meier curve of OS. Comparison between patients with single system disease vs. patients with multi system disease

**Fig. 4:**
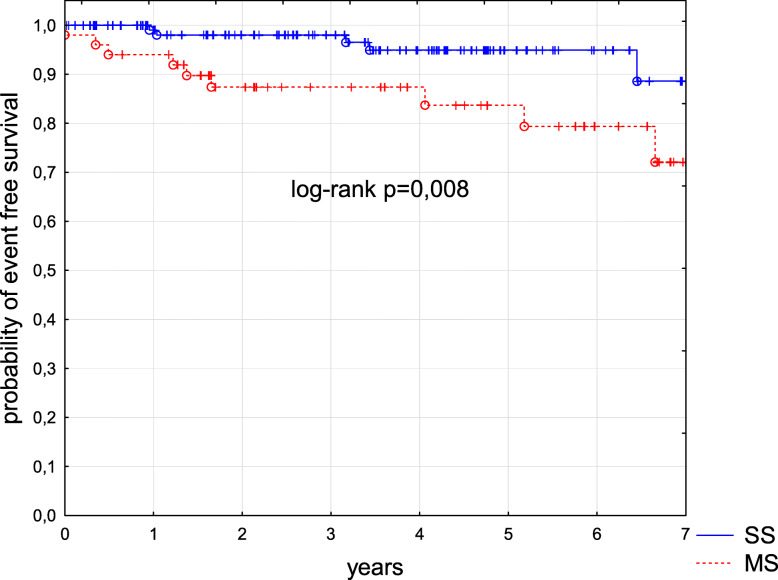
Kaplan-Meier curve of EFS for the study group. Comparison between patients with single system disease vs. patients with multi system disease

Six children were lost from the follow-up and three patients were still receiving treatment at the time of data collection. One hundred and seventy-one patients are alive with a median follow-up of 4.3 years from diagnosis (range from 0.1 to 18.1 years). Two children died, one infant prior to receiving treatment and one child due to a progressive disease 6 months post-diagnosis. Fourteen patients relapsed and are alive in second remission. Overall survival (OS) and event free survival (EFS) in the entire group were 0.99 and 0.91 respectively (Figs. 1 and 2). OS and EFS were significantly better in SS group as compared to MS group, p 0.03 and p 0.008, respectively (Figs. 3 and 4). Among 11 patients with BRAF mutation, one child relapsed and one progressed. The patient who relapsed was treated with second line chemotherapy (cytarabine and vincristine), while the patient who progressed received target therapy with vemurafenib. Both children are alive in complete clinical remission.

## Discussion

Langerhans cells histiocytosis (LCH) is a rare disease with unclear etiology. It may affect different age groups, but is most often observed in the first decade of life [1–4]. It can affect different organs and systems i.e. bones, skin, lymph nodes, liver, lung, spleen, hematopoietic system, and central nervous system (CNS). The spectrum of clinical manifestations is broad, from single-focus to life-threatening symptoms. Current classification divides LCH into two groups: single system (single organ or system is involved; found in about 55% of patients), and multisystem (involves 2 or more systems / organs; with or without risk organs involvement) [1–6]. In our study, the single system disease was found in 69% of patients, which may be associated with multisystem underestimation, since the total number of patients was not known (no data from 4 centers). Bones were most involved which is consistent with other reports [2, 4, 6].

The course of LCH can be diverse and often is unpredictable. It oscillates from spontaneous regression, through a long-term period of exacerbation of the disease, to an aggressive form rapidly leading to death. In 2010, Badalian-Very published the study describing the presence of mutations in the BRAF^V600E^ gene in histiocytes in patients with LCH [7]. Nowadays, it seems that the occurrence of mutations in the BRAF gene is usually associated with a more aggressive form of the disease, more frequent resistance to conventional chemotherapy, and with a higher probability of recurrence and progression [1, 6, 10–12]. For these reasons, molecular diagnostics is routinely carried out in many countries. In the present study, only 14% of patients underwent molecular tests and we strongly believe, it is necessary to introduce them for routine diagnostics in this group of patients in Poland.

The younger the child, the greater the risk of multisystem disease, and the greater the risk of the unfavorable course of LCH. In addition, the involvement of the risk organs (hematopoietic system, liver, spleen) is associated with a worse prognosis [1–4, 6]. In our material, both deceased patients had MS disease. What is more, first symptoms occurred before the age of 2 in both cases.

Although, the outcome of Polish patients can generally be considered as good, the need for the implementation of unified diagnostic procedures is clear. These must include molecular tests, treatment and other managements based on LCH guidelines. Furthermore, successful outcomes are dependent on the introduction of individualized aggressive local control measures. Thus, due to the rarity of appearances, a good inter-center cooperation is needed for a more thorough understanding of the nature of the disease, as well as for better treatment results.

## Conclusion

The treatment of LCH in Polish centers was effective. However, successful long-term outcomes are dependent on good diagnostic approaches and very judicious use of chemotherapy. Identifying areas for improvement is needed in order to generalize outcomes. The development of national cooperative groups to coordinate and optimize resources, adapt treatment guidelines, and develop centralized centers of excellence is imperative in the advancement of care for children with cancer worldwide.

## Data Availability

Data and material are available upon request. Katarzyna Drabko katarzynadrabko@umlub.pl

## Abbreviations

LCH: Langerhans cell histiocytosis
OS: Overall survival
EFS: Event free survival
SS: Single system disease
MS: Multi system disease
CNS: Central nervous system
